# miR-221 facilitates the TGFbeta1-induced epithelial-mesenchymal transition in human bladder cancer cells by targeting STMN1

**DOI:** 10.1186/s12894-015-0028-3

**Published:** 2015-04-28

**Authors:** Jun Liu, Jian Cao, Xiaokun Zhao

**Affiliations:** Department of Urology, 2nd xiangya Hospital, Central South University, NO.139 Middle Renmin Road, 410011 Changsha, Hunan China

**Keywords:** miR-221, Bladder cancer, EMT, STMN1, TGFβ1

## Abstract

**Background:**

Distant metastasis is the major cause of cancer-related death, and epithelial-to-mesenchymal transition (EMT) has a critical role in this process. Accumulating evidence indicates that EMT can be regulated by microRNAs (miRNAs). miR-221, as oncogenes in several human cancers, was significantly up-regulated in bladder cancers. However, the role of miR-221 in the progression of bladder cancer metastasis remains largely unknown.

**Methods:**

We used qRT-PCR and western blot to accurately measure the levels of miR-221, STMN1 and EMT markers in TGFβ1 induced EMT of bladder cancer cells. miR-221 inhibitors were re-introduced into bladder cancer cells to investigate its role on tumor metastasis which was measured by MTT, wound healing, transwell invasion and adherent assays. Luciferase reporter assay was used to reveal the target gene of miR-221.

**Results:**

miR-221 expression was greatly increased by TGFβ1 in bladder cancer cell. miR-221 inhibition reversed TGFβ1 induced EMT by sharply increasing the expression of the epithelial marker E-cadherin and decreasing the expression of the mesenchymal markers vimentin, Fibroactin and N-cadherin. Furthermore, miR-221 expression is positively correlated with malignant potential of bladder cancer cell through promoting loss of cell adhesion and prometastatic behavior. Luciferase reporter assay revealed that miR-221 negatively regulates STMN1 expression by direct targeting to the 3′UTR region of STMN1.

**Conclusions:**

Our study demonstrated that miR-221 facilitated TGFβ1-induced EMT in human bladder cancer cells by targeting STMN1 and represented a promising therapeutic target in the process of metastasis.

## Background

Bladder cancer is one of the most common worldwide malignancies. In developed countries, bladder cancer (BC) is the fifth most commonly diagnosed tumor and the second most common cause of death among genitourinary tumours [1]. So it is urgent to understand the molecular and cellular mechanisms of metastasis for investigating the development of bladder cancer. Currently, there is a theory considering Epithelial–Mesenchymal Transition (EMT) as the first step of metastasis [2]. Previous studies showed that EMT was a complex and reversible process initiated by specific substances so that epithelial cells gain mesenchymal characteristics in cervical and breast cancers [3-6]. Recent advances have fostered a more detailed understanding of molecular mechanisms and networks governing EMT in tumor progression [7]. Although several growth factors participate in EMT, TGFβ is the most studied. Upon TGFβ1 treatment, epithelial cell changed from a cuboidal to an elongated spindle shape with enhanced expressions of Snail1 & Twist1 and subsequently decreased expression of E-cadherin [8]. Accumulating studies showed that TGFβ could consequently promote cancer progression through the induction of EMT, during which tumor cells become more invasive and metastatic [9]. However, whether miRNA are involved in regulating TGFβ -induced EMT in BC remains obscure.

MicroRNA (miRNA), a class of naturally occurring, 17–25 nucleotide small noncoding small RNA, regulates the expression of genes through binding to the 3′ untranslated regions (3′ UTR) of target mRNAs. Recently, growing evidence suggests that aberrant expression of microRNAs (miRNAs) is a common phenomenon in bladder cancer and miRNAs can be key players in diverse physiological and pathological processes, such as embryonic development, tumorigenesis, metastasis, metabolism and apoptosis [10]. Recently, miRNAs have also been demonstrated to be involved in the process of epithelial–mesenchymal transition (EMT) by modulation of EMT-related genes [11]. MiR-7 reverses the EMT of breast cancer stem cells by downregulating the STAT3 pathway [12]. MicroRNA-451 induces EMT in docetaxel-resistant lung adenocarcinoma cells by targeting proto-oncogene c-Myc [13]. More interestingly, a recent study has shown that miRNA192 were upregulated by TGF- β 1 in mouse mesangial cells, and miRNA192 plays a pivotal role in diabetic nephropathy, mediated via controlling TGF-β1-induced collagen I expression by downregulating E-box repressors [14]. miRNA-200 and miRNA-205 were downregulated during TGF β mediated EMT and regulated EMT by targeting the E-cadherin transcriptional repressors ZEB1 and SIP1[15].

miR-221 has been shown to participate in both the onset and progression of various malignant tumors, including ovarian cancer [16,17]. For example, Qin J demonstrated that miR-221 is an oncogenic miRNA and regulates CRC migration and invasion through targeting reversion-inducing cysteine-rich protein with Kazal motifs (RECK) [18]. miR-221 is a critical modulator in the Hepatocellular carcinoma signaling pathway, and miR-221 silencing inhibits liver cancer malignant properties in vitro and in vivo [19]. Recent studies showed that Human micro-RNAs miR-221 was significantly up-regulated in bladder cancers [20]. Lu et al. revealed that miR-221 was significantly up-regulated in bladder cancer and miR-221 silencing predisposed T24 cells to undergo apoptosis induced by TRAIL [21]. However, to the best of our knowledge, the specific role of miR-221 in the TGFβ1-induced EMT in bladder cancer and the mechanisms underlying its effects remain unknown. Because EMT is of particular significance as a marker of tumor invasion and metastasis and TGFβ1 treatment represents a classical induction approach for in vitro EMT research, we believe that elaborating both the specific roles of miR-221 in TGFβ1-induced EMT models of bladder cancer and the latent molecular mechanisms will enlarge our theoretical understanding of human bladder cancer and provide future clinical approaches to treating this disease.

## Methods

### Cell culture and TGFβ1 treatment

Human bladder cancer cell lines (RT4 and T24) (Shanghai Cell Bank, China) were propagated in DMEM (Invitrogen) supplemented with 10% FCS at 37°C in 5% CO_2_ cell culture incubator. In the TGFβ1 (Sigma Aldrich, St. Louis, MO) treatment, the cells were serum starved overnight and treated with 2.5 ng/ml TGFβ1 for 24 hours. The medium containing TGFβ1 was replaced every 24 hours (The Clinical Research Ethics Committee of Central South University approved the research protocols, and written informed consent was obtained from the participants).

### microRNA and transient transfection

miR-221 mimics, control mimics, miR-221 inhibitors, and control inhibitors were purchased from RiboBio (Guangzhou, China). RT4 and T24 cells were seeded into 6-well plates until 50%–60% confluent and then transiently transfected with 60 nM control or miR-221 mimics or with 120 nM control or miR-221 inhibitors using the X-treme GENE siRNA Transfection Reagent (Roche, Indianapolis, IN, USA) according to the manufacturer’s instructions. After 48 hours of miRNA transfection, the cells were harvested for further study.

### Quantitative real-time PCR

Total RNA was isolated using TRIzol reagent (Invitrogen, Carlsbad, CA, USA) according to the manufacturer’s recommendations. For mRNA detection, first-strand cDNA was synthesized using a PrimeScript RT reagent kit (Perfect Real Time; Takara, Dalian, China). Quantitative real-time PCR was performed using a SYBR Premix Ex Taq™ II kit (Takara, Dalian, China) on a CFX96 real-time PCR system (Bio- Rad, Hercules, CA, USA). The PCR conditions were as follows: 95°C for 30 s, followed by 40 cycles of 95°C for 5 s and 60°C for 34 s. β-Actin was used as an internal control to normalize the results. For miRNA detection, miR-221 levels were determined using a TaqMan microRNA kit (Applied Biosystems) and normalized to small nuclear RNA (Rnu6), which served as a control; the data were expressed as the log 2 fold change in respective miR/U6 snRNA levels. Primers for miR-221 and U6 reverse transcription and amplification were designed by and purchased from RiboBio Co., Ltd. (Guangzhou, China).

### Western blot analysis

Whole cell extracts were prepared with a cell lysis reagent (Sigma-Aldrich, St. Louis, MO, USA) according to the manual, and then, the protein was quantified by a BCA assay (Pierce, Rockford, IL, USA). Then, the protein samples were separated by SDS-PAGE (10%) and detected by Western blot using polyclonal (rabbit) anti-STMN1, anti-Fibroactin, anti-N-Cadherin, anti-E-Cadherin and anti-Vimentin antibody (Santa Cruz Bio-technology, Santa Cruz, CA, USA, 1:1000). Goat anti-rabbit IgG (Pierce, Rockford, IL, USA) secondary antibody conjugated to horseradish peroxidase and ECL detection systems (SuperSignal West Femto, Pierce) were used for detection.

### Cell survival assay

The 3-(4,5-dimethylthiazal-2-yl)-2,5-diphenyl-tetrazolium bromide (MTT) assay was used to estimate cell viability [22]. Briefly, cells were plated at a density of 1 × 10^4^ cells per well in 96-well plates. After exposure to specific treatment, the cells were incubated with MTT at a final concentration of 0.5 mg/ml for 4 h at 37°C. After the removal of the medium, 150 mM DMSO solutions were added to dissolve the formazan crystals. The absorbance was read at 570 nm using a multi-well scanning spectrophotometer reader. Cells in the control group were considered 100% viable.

### Invasion assay

Cells were cultivated to 80% confluence on the 12-well plates. Then, we observed the procedures of cellular growth at 24 h. All the experiments were repeated in triplicate. The transwell invasion chambers were used to evaluate cell invasion. Then cells invasing cells across the membrane were counted under a light microscope.

### Adhesion assay

Cells were pretreated with or without different concentrations of excisanin A for 24 h. The cells were suspended in serum-free DMEM medium to form a single-cell suspension and were seeded into 96-well plates precoated with Matrigel™ (BD Biosciences, Franklin Lakes, NJ, USA). The wells were incubated at 37°C for 50 min and washed three times with PBS to remove the non-adherent cells. Cell viability was determined via the MTT assay described above.

### Wound healing assay

For the wound healing assay, cells were seeded in 12-well plates and grown to 90% confluence. Monolayers in the center of the wells werescraped with pipette tips and washed with PBS. Subsequently, the cellswere cultured in serum-free DMEM medium in the absence or presenceof different concentrations of excisanin A for 24 h. Cell movement intothe wound area was monitored and photographed at 0 and 24 h usinga light microscope. The migration distance between the leading edge ofthe migrating cells and the edge of the wound was compared as previous work [23].

### Luciferase reporter assay

HEK293 cells (1 × 10^4^ cells/well) were plated in a 48-well plate and cotransfected with 50 n M of either miR-221 or microRNA control (miRcontrol), 20 ng of either pGL3-STMN1-3′-UTR-WT or pGL3-STMN1-3′-UTR-Mutation, and 2 ng of pRL-TK (Promega, Madison, WI, USA) using Lipofectamine TM 2000 (Invitrogen). The pRL-TK vector was cotransfected as an internal control to correct the differences in both transfection and harvest efficiencies. HEK293 cells were collected 48 h after transfection and assays were performed by using the dual luciferase reporter assay system (Promega).

### Statistical analysis

All experiments were performed at least in triplicate, and each experiment was independently performed at least 3 times. Data are presented as the means ± standard deviation (SD) and were analyzed using SPSS 19.0. Statistical significance was assessed using the two-tailed unpaired Student’s t-test. Differences were considered statistically significant when the P value was <0.05.

## Results

### MiR-221 and expression in TGFβ1-induced EMT

**Figure 1:**
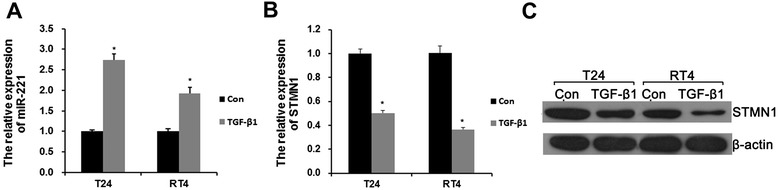
The expression level of miR-221 and STMN1 before/after TGFβ1 treatment. **A**, qRT-PCR analysis indicated miR-221 expression in TGFβ1-induced EMT in both RT4 and T24 cells. qRT-PCR analysis **(B)** and western blot analysis **(C)** indicated STMN1 expression in TGFβ1-induced EMT in both RT4 and T24 cells. Error bars represent ± S.E. and *p < 0.05 versus control.

In preliminary experiments, we tested various TGFβ1 concentrations and incubation durations for their ability to induce EMT in RT4 and T24 cells. Based on these experiments, we determined the dose of 2.5 ng/ml and the duration of 24 hours as appropriate conditions for EMT stimulation by TGFβ1. To explore whether miR-221 is involved in TGFβ1-induced EMT in human bladder cancer cells, we first attempted to determine the expression level of miR-221 before/after TGFβ1 treatment. Surprisingly, compared with control group, miR-221 expression was significantly increased in both RT4 and T24 cells incubated with TGFβ1(Figure 1A). As shown in Figure 1B and C, STMN1 was significantly decreased by TGFβ1 treatment at both the mRNA and protein levels. These results suggested that miR-221 and STMN1 involved in TGFβ1-induced EMT of bladder cancer cells.

### STMN1 is negatively regulated by MiR-221 in TGFβ1-induced EMT

**Figure 2:**
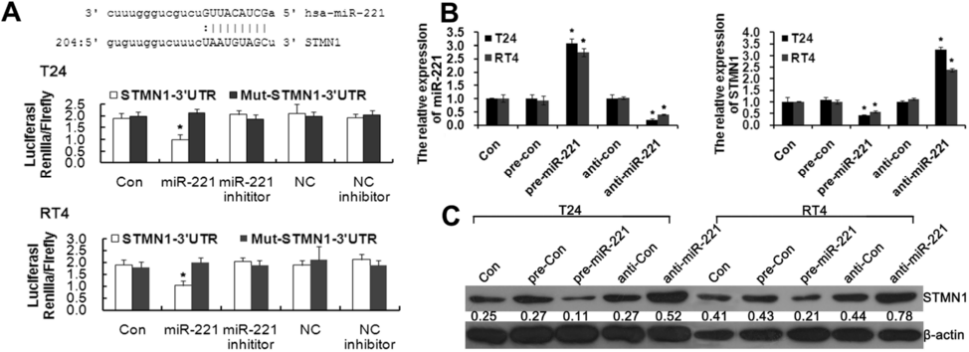
miR-221 negatively regulates STMN1 expression. **(A)** Luciferase reporter assay with co-transfection of wild-type or mutant STMN1 and miR-221 mimics or miR-221 inhibitor or negative–control or inhibitor negative-control or blank control in H9c2 cells. **(B)** qRT-PCR analyses were performed to examine the effects of miR-221 on expression of STMN1. **(C)** Western blotting was performed to determine effects of miR-221 on expression of STMN1 protein in T24 and RT4 cells. Error bars represent ± S.E. and *p < 0.01 versus control.

We performed a bioinformatic analysis using mirco-RNA.org and predicted that STMN1 was the possible target gene of miR-221. To confirm this speculation, 3′UTR luciferase reporter assay was used in this study. As shown in Figure 2A, co-transfection of miR-221 suppressed the luciferase activity of the reporter containing wild-type STMN1 3′ UTR sequence, but failed to inhibit that of mutated STMN1 by dual-luciferase reporter assay. These result suggested that miR-221 directly binds to the STMN1 3′UTR. Furthermore, we employed miR-221 mimics and inhibitors to specifically overexpress and knock down the endogenous expression of miR-221 in RT4 and T24 cells. As shown in Figure 2B and C, STMN1 expression was significantly decreased by transfection with miR-221 mimics and was greatly increased by transfection with miR-221 inhibitors at both the mRNA and protein level. Therefore, miR-221 negatively regulates STMN1 expression in bladder cancer cells. Together, these results demonstrated that miR-221 directly binds to its complementary sequence motif in the STMN1 3′UTR, thus negatively regulating STMN1 expression.

### MiR-221 expression is positively correlated with malignant potential of bladder cancer cell

**Figure 3:**
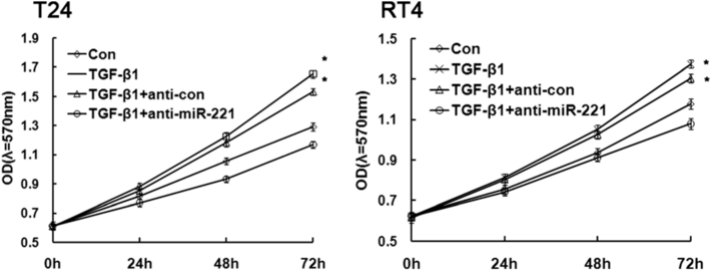
Cell survival curve was measured by MTT. MTT analysis revealed the effects of miR-221 on cell survival in TGFβ1-induced EMT of bladder cancer. Error bars represent ± S.E. and *p < 0.05 versus control.

Enhanced cellular malignant capacity, including cell survival, migration and invasion abilities, is the functional hallmarks of an EMT process. Because incubation with TGFβ1 resulted in increasing miR-221 levels in both RT4 and T24 cells, we silenced miR-221 to test whether miR-221 is involved in motility changes in bladder cancer cells, aiming to examine the specific role of miR-221 in TGFβ1-induced EMT. The cell line RT4 and T24, which constitutively expresses high levels of miR-221, was transfected with miR-221 siRNA to knock down its endogenous miR-221 expression or with a scrambled siRNA as a control. As shown in Figure 3, MTT results showed that significantly increase of cell survival was observed in TGFβ1 group compared with control group and significantly attenuation of cell invasion was observed in TGFβ1 + anti-miR-221 group compared with TGFβ1 group and TGFβ1 + anti-Con group. These result indicated that TGFβ1 greatly promoted the cell survival in bladder cancer, and TGFβ1-induced cell survival was reversed by miR-221 inhibition.

**Figure 4:**
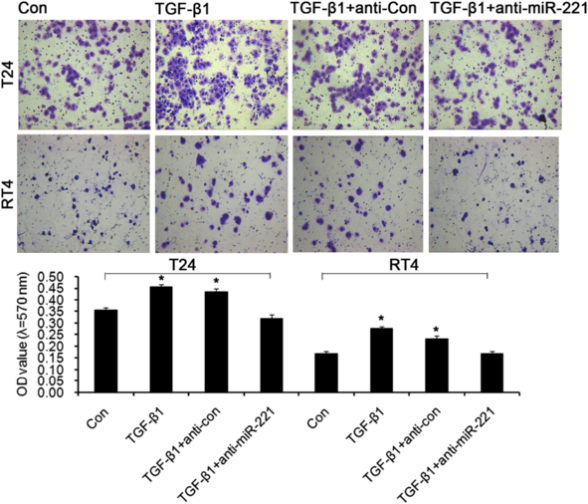
Transwell invasion assay revealed the effects of miR-221 on cell invasion in TGFβ1-induced EMT of bladder cancer. Error bars represent ± S.E. and *p < 0.05 versus control.

**Figure 5:**
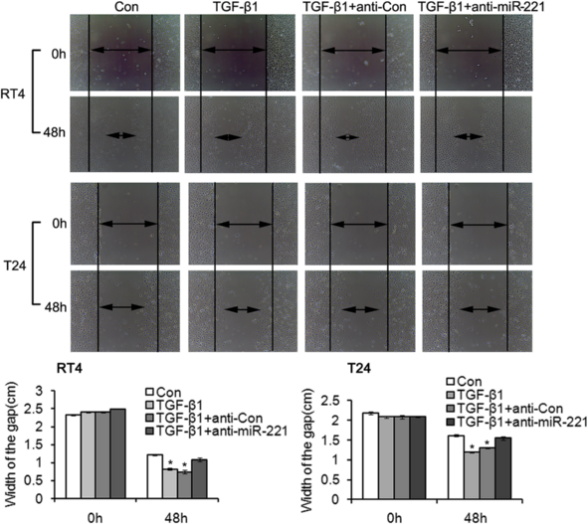
Wound healing assay revealed the effects of miR-221 on cell migration in TGFβ1-induced EMT of bladder cancer. Error bars represent ± S.E. and *p < 0.05 versus control.

**Figure 6:**
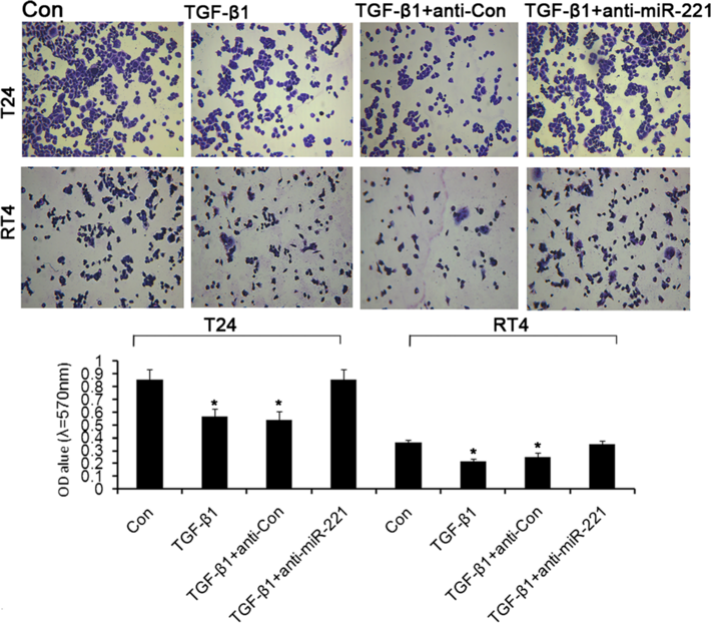
Adhesion assay revealed the effects of miR-221 on cell adhesion in TGFβ1-induced EMT of bladder cancer. Error bars represent ± S.E. and *p < 0.05 versus control.

Transwell invasion assay showed that significant increase of bladder cancer cell invasion was observed in TGFβ1 group compared with control group and significant attenuation of bladder cancer cell invasion was observed in TGFβ1 + anti-miR-221 group compared with TGFβ1 group and TGFβ1 + anti-Con group. These results indicated that TGFβ1-induced cell invasion was reversed by miR-221 inhibition (Figure 4). As shown by the representative images presented in Figure 5, wound healing generated results that were similar to those obtained using the transwell assay. The cell migration rates of cells in miR-221 inhibition groups were shown to be significantly lower than control group, as evaluated using a wound-healing assay. In contrast, miR-221 inhibition decreased the migration and invasion of bladder cancer cells, perhaps through the reversal of EMT. In conclusion, miR-221 plays an important role in mediating the malignant potential of metastatic bladder cancer cells. In conclusion, these results demonstrated that miR-221 promoted the migration and invasion of bladder cancer cells, possibly through the induction of EMT. Cell adherent assay (Figure 6) generated results that were similar to those obtained using the transwell migration assay, suggesting that inhibition of miR-221 resulted in an attenuation of TGFβ1-induced invasion and adherent capacity.

### MiR-221 inhibition attenuated TGFβ1-induced EMT in bladder cancer cells

**Figure 7:**
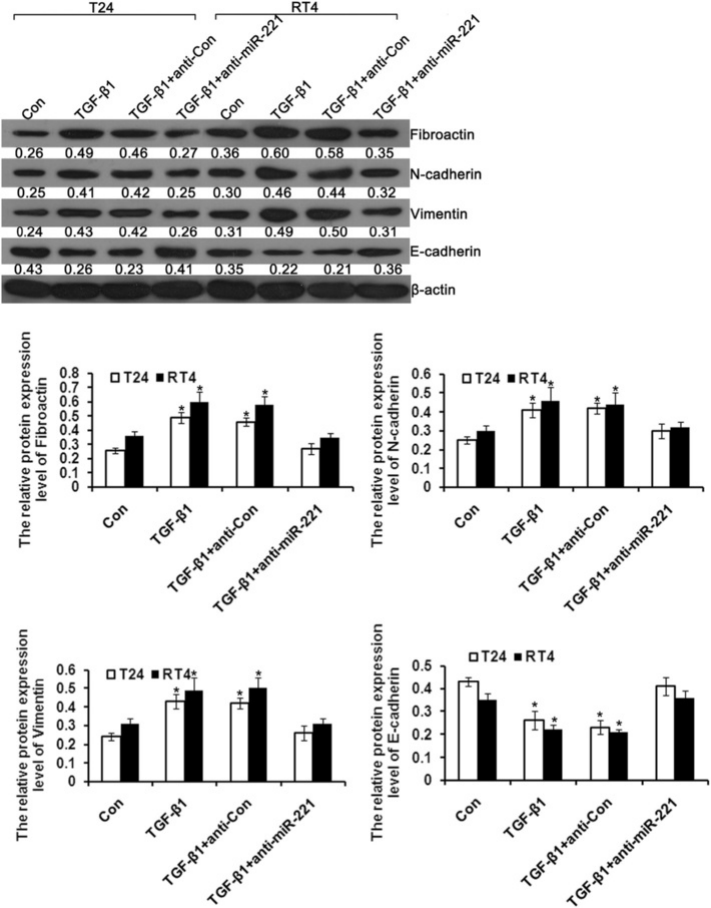
Western blot analysis showed differences in epithelial and mesenchymal markers between cells treated with TGFβ1 alone and cells treated with both TGFβ1 and miR-221 inhibitor. Error bars represent ± S.E. and *, p < 0.05 versus control.

To further assess the effects of miR-221 on TGFβ1-induced EMT, we transfected control and miR-221 inhibitor into T24 and RT4 cells, and then cells were treated with TGFβ1, and detected the effects of miR-221 on TGFβ1-induced EMT in bladder cancer cells. Western blotting analyses (Figure 7) showed that TGFβ1 treatment sharply decreased the expression of the epithelial marker E-cadherin and increased the expression of the mesenchymal markers vimentin, Fibroactin and N-cadherin. However, combination of miR-221 inhibition and TGFβ1 reversed the suppression of epithelial genes and the upregulated expression of mesenchymal genes compared to treatment with TGFβ1 alone. In conclusion, miR-221 inhibition attenuated TGFβ1-induced EMT in bladder cancer cells.

## Discussion

In the present study, we found that miR-221 expression is specifically upregulated in TGFβ1-responsive bladder cancer cells and contributes significantly to the development of the EMT phenotype. The EMT is characterized by changes in morphology, the loss of intercellular junctions, increased motility, decreased proliferation, and alterations in gene expression. Our study demonstrated that miR-221 knockdown inhibits these TGFβ1-induced changes. Similarly to our observations, miR-221 is also found to promote EMT in other types of human cancer cells, such as breast cancer cells [24,25]. Taken together, these findings confirm the involvement of miR-221 in TGFβ1-mediated EMT. Moreover, dysregulation of miR-221 is frequently found in various human cancers, including ovarian cancer, prostate cancer, endometrial cancer, and breast cancer, and is associated with features of cancerous progression and metastasis [23]. In our study, miR-221 inhibition decreased cell survival, migration and invasion capacities and enhanced adhesion capacities in bladder cancer cells. In conclusion, these results demonstrated that the expression of miR-221 is positively correlated with bladder cancer cell metastasis and further corroborate the connection between miR-221 expression and EMT features in human bladder cancer cells. Therefore, targeting miR-221 might represent a feasible and attractive option for the future clinical treatment and prevention of human bladder cancer.

Recently, accumulating evidence indicated that miRNAs play key roles in carcinogenesis by modulating gene expression on posttranscriptional level. Our data further support this conclusion that upregulated miR-221 suppressed expression of STMN1 in bladder cancer cells, further promoted the progressive and metastatic potential of human bladder cancer. The microtubule-destabilising protein, stathmin 1/oncoprotein 18 (STMN1), has an important role during mitosis, influencing cell cycle progression [26,27]. In addition, STMN1, as an oncoprotein, is involved in tumour metastasis, cell invasion and migration [28-30] and is a considered therapeutic cancer target [31]. Recently, Williams K, et al showed that inactivation of STMN1 is key to promoting oncogenesis and EMT [32]. loss-of-STMN1 compromises cell-cell adhesion, which is followed by EMT, increased cell migration, and metastasis via cooperative activation of p38 and through TGF-β1-independent and -dependent mechanisms [32]. In this study, STMN1 was downregulated by TGFβ1 in bladder cancer. By employing the Dual Luciferase Reporter Assay System, we report for the first time that miR-221 suppressed STMN1 expression by targeting STMN1 3′UTR in bladder cancer. Collectively, these findings confirm that miR-221 plays a significant role in cancer development and progression by directly targeting STMN1.

## Conclusion

Taken together, our findings demonstrate for the first time that miR-221 can facilitate the TGFβ1-induced EMT process in human bladder cancer cells by suppressing STMN1. Moreover, miR-221 levels are also correlated with pathological mesenchymal behaviors, such as decreased cell adherent capacity and increased cell survival, migration and invasion in human bladder cancer cells. Further studies targeting STMN1 and the mechanism of miR-221 regulation by TGFβ1 induction will provide promising and feasible options for the treatment and prevention of human bladder cancer.

## Abbreviations

TGFβ1: Transforming growth factor beta 1
STMN1: Stathmin 1
EMT: Epithelial-to-mesenchymal transition
BC: Bladder cancer
